# Identification of a human TFPI-2 splice variant that is upregulated in human tumor tissues

**DOI:** 10.1186/1476-4598-6-20

**Published:** 2007-03-12

**Authors:** Prakasha Kempaiah, Hitendra S Chand, Walter Kisiel

**Affiliations:** 1 Department of Pathology, University of New Mexico Health Sciences Center, Albuquerque, NM, USA

## Abstract

**Background:**

Previous studies have shown that the expression of tissue factor pathway inhibitor-2 (TFPI-2), a matrix-associated Kunitz-type serine proteinase inhibitor, is markedly down-regulated in several tumor cells through hypermethylation of the TFPI-2 gene promoter. In the present study, RT-PCR analysis of total RNA from both human normal and tumor cells revealed a novel 289 nucleotide splice variant of the TFPI-2 transcript designated as aberrantly-spliced TFPI-2 (asTFPI-2).

**Results:**

Nucleotide sequence analyses indicated that asTFPI-2 consists of complete exons II and V, fused with several nucleotides derived from exons III and IV, as well as six nucleotides derived from intron C. 5'- and 3'-RACE analyses of total RNA amplified exclusively the wild-type TFPI-2 transcript, indicating that asTFPI-2 lacks either a 5'-untranslated region (UTR) or a 3'-poly (A)^+ ^tail. Quantitative real-time RT-PCR analyses revealed that several human tumor cells contain 4 to 50-fold more copies of asTFPI-2 in comparison to normal cells. In spite of the absence of a 5'-UTR or poly (A)^+ ^tail, the asTFPI-2 variant exhibited a half-life of ~16 h in tumor cells.

**Conclusion:**

Our studies reveal the existence of a novel, aberrantly-spliced TFPI-2 transcript predominantly expressed in tumor cells and provides suggestive evidence for an additional mechanism for tumor cells to down-regulate TFPI-2 protein expression enhancing their ability to degrade the extracellular matrix.

## Background

Tissue factor pathway inhibitor-2 (TFPI-2) is a 32 kDa Kunitz-type serine proteinase inhibitor synthesized by a variety of cells and directionally secreted into their extracellular matrix (ECM) where it is thought to regulate plasmin-mediated ECM degradation and remodeling (reviewed by Chand et al. [1]). As matrix degradation is an important step in tumor invasion and metastasis, several, but not all, tumor cells downregulate TFPI-2 expression [2,3]. In this regard, overexpression of TFPI-2 in several tumor cells was shown to inhibit their growth, invasiveness, angiogenic potential and metastatic potential [4-9]. The mechanism whereby some tumor cells downregulate TFPI-2 synthesis has been primarily attributed to transcriptional silencing through hypermethylation of CpG sites in the TFPI-2 promoter [10-14], inasmuch as treatment of these tumor cells with a methyltransferase inhibitor, 5'-aza-2'-deoxycytidine, restored TFPI-2 transcription[14]. In addition, several highly aggressive tumors delete the locus for the TFPI-2 gene in the chromosome 7q region [15-17], resulting in the total loss of TFPI-2 protein expression in these cells. Accordingly, the TFPI-2 gene is becoming increasingly recognized as a tumor suppressor gene, since its down-regulation in several types of cancers allow for enhanced tumor growth and metastasis.

In view of its apparent role in cancer progression, we initiated a study to quantify TFPI-2 transcript levels in total RNA samples from selected normal human tissue, as well as their corresponding tumor tissue. In the course of these studies, we detected a novel, aberrantly-spliced variant of TFPI-2 mRNA derived from TFPI-2 pre-mRNA splicing at exon/intron boundaries, as well as at new sites within exons and introns. The levels of the aberrantly-spliced variant of TFPI-2 were either very low or undetectable in normal tissue, but markedly upregulated in tumor tissues and several tumor cell lines. These findings provide suggestive evidence for an additional mechanism for tumor cells to down-regulate TFPI-2 expression through aberrant splicing.

## Results

### Novel TFPI-2 splice variant generated by aberrant splicing

**Figure 1:**
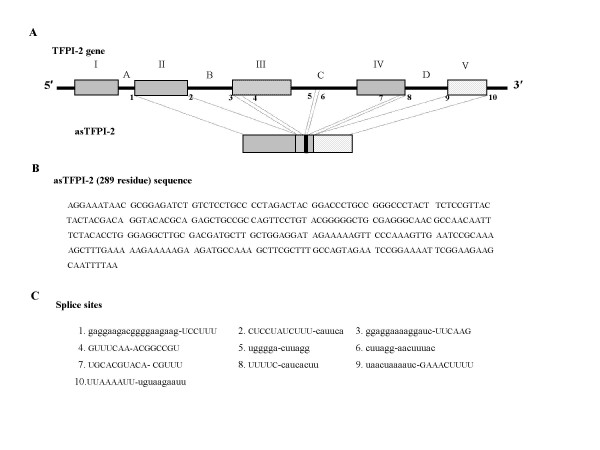
**A schematic representation of the full-length TFPI-2 gene and a novel TFPI-2 splice variant**. (A) The full-length TFPI-2 gene consists of 5 exons (designated by roman numerals) and 4 introns (designated by letters). (B) The novel splice variant reported here is generated by splicing of complete exon II, 15 nucleotides of exon III (AAAGTTCCCAAAGTT), 6 nucleotides of (GAATCC) intron C, 7 nucleotides of exon IV (GCAAAAG) and complete exon V. (B) The complete (289 bp) asTFPI-2 cDNA sequence is shown in 5' to 3' orientation. (C) The nucleotide sequences at the splice site junctions that result in the generation of asTFPI-2. The arabic numbers in the TFPI-2 gene diagram (A) correspond to the splice site locations. Splice site numbers 1–3 and 8–9 have normal consensus splice sites, whereas sites 4–7 and 10 are non-consensus splice sites. The intronic sequences are presented in lower case letters, while the exonic sequence is presented in upper case letters.

In preliminary studies designed to assess the levels of TFPI-2 transcripts in various normal and tumor tissues, co-amplification of a lower molecular weight cDNA was observed following RT-PCR of total RNA. The low *M*_*r *_cDNA was faintly visible in normal tissues (lung, colon and liver), but was markedly upregulated in the corresponding tumor tissues. Nucleotide sequence analyses of the low *M*_*r *_cDNA amplified from the total RNA of lung tumor tissue revealed a novel, 289 nucleotide, aberrantly-spliced form of the TFPI-2 transcript designated as asTFPI-2 (Fig. 1B). Subsequent studies revealed that the nucleotide sequence of the low *M*_*r *_cDNA from HepG2 cells was identical to that observed in lung tumor tissue (data not shown). Both 5' and 3' RACE analyses of total RNA derived from several tissues and cell lines tested resulted exclusively in the amplification of the normal TFPI-2 transcript. In these RACE analyses, several attempts were made to identify any 5' and 3'-untranslated regions (UTRs) by varying reaction conditions and using different sets of primers, but each attempt only amplified the 5' and 3' ends of normal TFPI-2 (data not shown). Moreover, the 5' and 3' boundaries of the asTFPI-2 were also assessed by primer walking studies using a series of primer combinations spanning the entire regions of exon I, intron A and the 3' UTR (Fig. 1). Thus, these results indicate that the aberrantly-spliced asTFPI-2 reported here lacks any unique 5' and 3'-UTRs and consists of complete exons II and V, fused with 14 nucleotides derived from exon III, 7 nucleotides derived from exon IV, and 6 nucleotides of intron C (Fig. 1A).

### The levels of asTFPI-2 are elevated in tumor tissues and tumor cell lines

**Figure 2:**
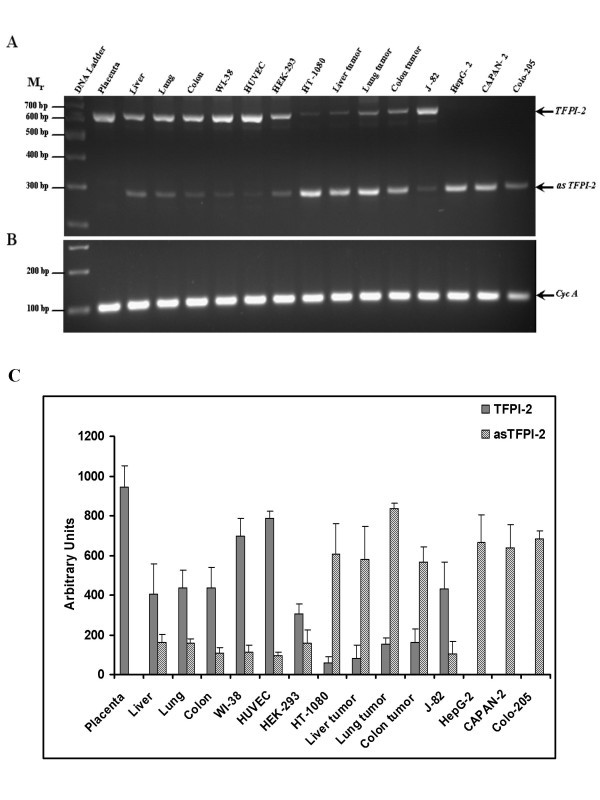
**Relative quantitation of human TFPI-2 transcripts in various normal and tumor cells**. Agarose gel electrophoresis analysis of RT-PCR products from various cells and tissues. Panel A depicts TFPI-2 (622 bp) and asTFPI-2 (289 bp) transcripts, while panel B depicts cyclophilin A transcripts. The data presented in panel C represents the amount of transcripts normalized to 10,000 arbitrary units of cyclophilin A amplified from each sample. Each column represents the average of two amplification reactions (error bars represent standard deviation) performed on a single cDNA sample reverse-transcribed from RNA derived from each sample.

An apparent variation in the transcripts levels of TFPI-2 and asTFPI-2 from primary cells and tumor cells was observed by semi-quantitative RT-PCR, and subsequently validated by quantitative real-time RT-PCR analysis. Semi-quantitative RT-PCR was carried out using common set of primers to amplify TFPI-2 and asTFPI-2 transcripts in a single reaction (Fig. 2A). The relative intensity data of each amplicon derived was normalized using the cyclophilin A transcript (Fig. 2B). The quantiscan analysis of each amplicon indicated, on average, that there are ~10 fold more TFPI-2 copies in normal tissue in comparison to tumor tissues, and that TFPI-2 is absent in many tumor cell lines (HepG2, Capan-2 and Colo-205). In contrast, with the exception of the J82 bladder carcinoma cell line, there are 7-fold more copies of the asTFPI-2 splice variant in all tumors compared to their corresponding normal tissues or normal cultured cell lines (Fig. 2C).

**Figure 3:**
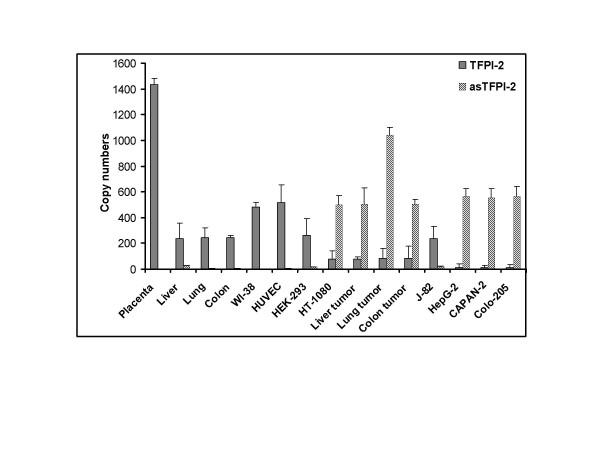
**Quantitative real-time RT-PCR analyses of human TFPI-2 transcripts in normal and tumor samples**. The copy numbers of TFPI-2 and asTFPI-2 in several selected samples were determined and normalized to 10^2 ^copies of cyclophilin in each sample. The copy numbers were interpolated from standard curves correlating the threshold cycle number (C_t _values) and copy numbers of TFPI-2 and asTFPI-2. Each column represents the average of three amplification reactions (error bars represent standard deviation) performed on a single cDNA sample reverse-transcribed from RNA derived from each sample.

Quantitative real-time RT-PCR analyses were performed separately for TFPI-2 and asTFPI-2 using specific primers (see Materials and methods). The copy numbers for TFPI-2, asTFPI-2 and cyclophilin A transcripts were derived from standard curves of their respective plasmids. The copy numbers of both TFPI-2 and asTFPI-2 observed were normalized to 10^2 ^copy numbers of cyclophilin A in respective samples (Fig. 3). A strong linear relationship between the C_t _and the log of the number of copies was consistently demonstrated (R^2 ^≥ 0.99), and melting curves confirmed the presence of a single amplicon. The data analyses of results from three independent measurements revealed highly variable amounts of TFPI-2 and asTFPI-2 transcripts between normal and tumor cells, but on average, there are 10-fold more TFPI-2 copies in normal tissue compared to tumor tissues, whereas there are 53-fold more copies of asTFPI-2 splice variant in tumor tissue or tumor cell lines compared to normal tissues or cultured cell lines. In general, normal tissues and cell lines contained higher levels of full-length TFPI-2 while tumor tissues and tumor cell lines, with the exception of the bladder carcinoma J82 cell line, contained 4–50 fold higher levels of the asTFPI-2 transcripts. In the case of placenta, asTFPI-2 transcripts were undetectable. The data presented in Figures 2 and 3 reflect an average of three independent experiments.

### Stability of asTFPI-2

**Figure 4:**
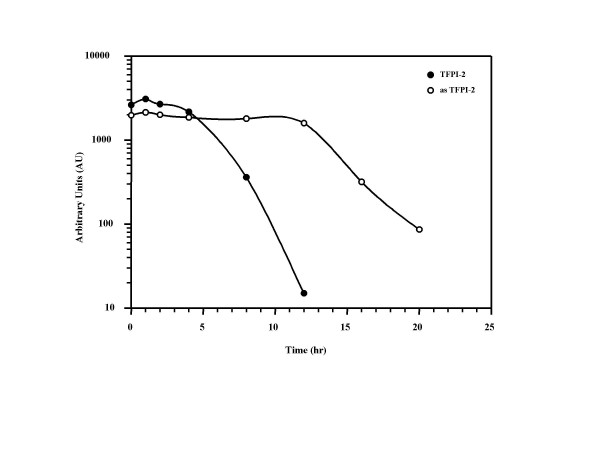
**Stability of TFPI-2 and asTFPI-2 transcripts**. HUVECs and Colo-205 cells were treated with 5 μM actinomycin D for various durations to block transcription. Total RNA was extracted at the indicated time points following the addition of actinomycin D, and quantitative real-time RT-PCR amplification were performed as described in the text to determine copy numbers of each TFPI-2 mRNA. Each data-point represents the average of three amplification reactions performed on a single cDNA sample reverse-transcribed from RNA derived from each sample.

It has been shown in numerous reports that actinomycin D inhibits cell proliferation by forming a stable complex with single-stranded DNA and blocking the movement of RNA polymerase that interferes with DNA-dependent RNA synthesis [18]. To assess the stability of the full-length TFPI-2 and asTFPI-2 transcripts, the transcription inhibitor actinomycin D was added to HUVEC and Colo-205 cells, and the total RNA was extracted at selected intervals. The copy numbers of the TFPI-2 and asTFPI-2 transcript were measured by real-time RT-PCR in order to compare the rate of mRNA decay in these two cell lines (Fig. 4). As shown in Fig 4, a half-life value of ~16 h was obtained for asTFPI-2 in a colon carcinoma cell line, in comparison to ~8 h for TFPI-2 mRNA in HUVECs. At this point, the functional significance of these relative values is difficult to interpret, given that they were obtained in different cell lines out of necessity due to the lack of a single cell line that produces each of these transcripts in sufficient quantities. Nonetheless, these findings provide suggestive evidence that the half-life of asTFPI-2 may be significantly longer than TFPI-2 mRNA in cultured cells.

### Northern blot analysis

**Figure 5:**
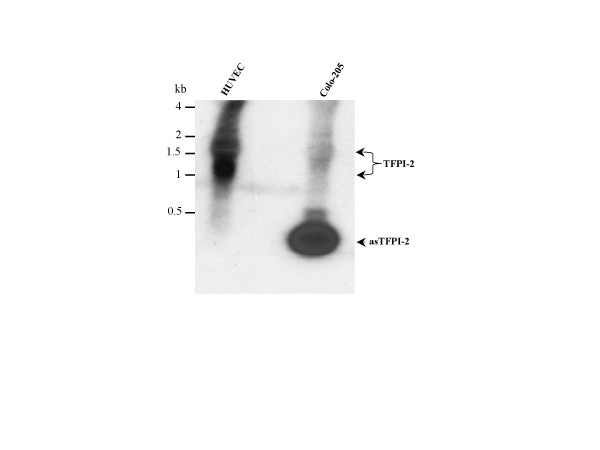
**Northern blot analysis of human TFPI-2 transcripts in normal and tumor cells**. Total RNA from HUVEC and Colo-205 cells was extracted and equal amounts (20 μg) of RNA was electrophoresed in a 1.2% agarose-formaldehyde gel and subsequently transferred to a nylon membrane and probed with ^32^P-asTFPI-2 cDNA. RNA molecular weight markers are indicated on the left. The probe detected both major (1.2 kb) and minor (1.8 kb) TFPI-2 transcripts in the case of HUVECs, and approximately a 300 bp asTFPI-2 transcript in Colo-205 cells.

To confirm the presence of TFPI-2 and asTFPI-2 transcripts in a normal (HUVEC) and a tumor (Colo-205) cell line, we performed Northern hybridizations using an asTFPI-2 specific cDNA radiolabeled probe. The analysis revealed a major 1.2-kb transcript as well as a minor 1.8-kb transcript in HUVECs (Fig. 5), as reported earlier [19]. TFPI-2 transcript was not detectable in Colo-205 cells, whereas the asTFPI-2 (~300 bp) transcript was readily observed in these cells (Fig. 5).

### TFPI-2 protein levels in normal and tumor cell lines

**Figure 6:**
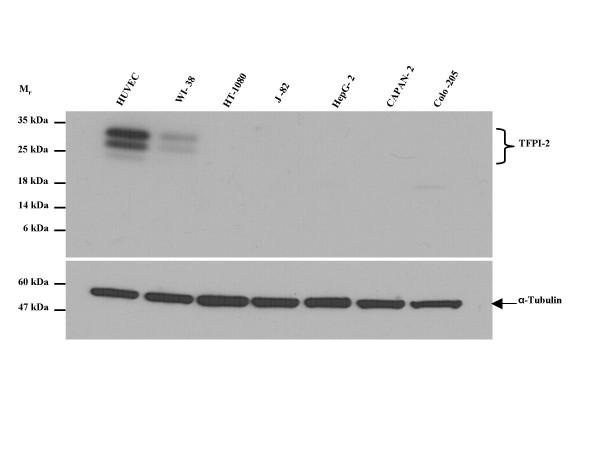
**Immunoblot analysis of TFPI-2 expression in normal and tumor cell lines**. Cell lysate samples were analyzed by immunoblotting using a polyclonal rabbit antibody raised against recombinant human α-TFPI-2 (upper panel). Alpha-tubulin expression (lower panel) was also assessed in the same lysate samples to establish equivalent loading volumes.

Despite the apparent absence of 5' and 3'-UTRs in asTFPI-2, we could not rule out the possibility that the asTFPI-2 transcript was translated within tumor cells by some novel mechanism, and that the putative translated product played some role in tumor biology. Accordingly, expression of TFPI-2 immunoreactive protein in lysates of two normal and five cancer cell lines was analyzed by immunoblotting using an anti-TFPI-2 polyclonal antibody raised against recombinant human α-TFPI-2 (Fig. 6). As expected, TFPI-2 protein was detected in HUVECs and WI-38 human fibroblast cell lysates, and migrated with the characteristic triplet pattern representing α, β and γ differentially glycosylated forms of TFPI-2 [20]. However, no immunoreactive protein was observed at the expected *M*_*r *_of ~11 kDa for asTFPI-2 in any of the cell lines examined (Fig. 6). It is also noteworthy that no immunoreactive protein was observed in J82 cells lysates, despite the relatively high copy numbers of TFPI-2 observed in these cells by quantitative real-time RT-PCR (Fig. 3). In addition, using primers specific for the 5'-UTR and poly (A)^+ ^tail of TFPI-2, a full-length transcript for TFPI-2 was observed in J82 cells (data not shown). Precisely why J82 cells do not synthesize TFPI-2 protein will require additional studies, but it is conceivable that the full-length TFPI-2 transcript in these cells contains one or more mutations that precludes translation.

## Discussion

Human TFPI-2, an ECM-associated Kunitz-type serine proteinase inhibitor [21], is thought to play a significant role in the regulation of plasmin-mediated ECM degradation during tumor cell invasion and metastasis, wound healing, and angiogenesis. Proteolytic degradation of the extracellular matrix, one of the key events in the process of tumor invasion and metastasis, depends on a delicate balance of matrix-degrading proteinases and their inhibitors. The TFPI-2 transcript is abundant in the full-term placenta, and is also present at various levels in several adult human tissues, such as liver, lung, skeletal muscle, heart, kidney, and pancreas [22]. Conversely, TFPI-2 mRNA levels fall with increasing malignancy in the case of breast, gastric, colon, pancreatic, laryngeal, renal, endometrial, glial and several other human malignant tumors [3,23,24].

In experiments designed to quantify TFPI-2 transcripts in human normal and corresponding tumor tissues using RT-PCR, we observed two TFPI-2 amplicons, using exon II (sense) and exon V (anti-sense) primers. Nucleotide sequencing analysis of both amplicons revealed that the larger *M*_*r *_amplicon (622 bp) corresponded to the full-length TFPI-2 cDNA, while the smaller *M*_*r *_cDNA (289 bp) was an aberrantly-spliced form of the TFPI-2 transcript, which we have provisionally designated as asTFPI-2. Primer walking with a series of primer combinations spanning the entire regions of exon I, intron A and 3' UTR revealed that the complete asTFPI-2 transcript consisted of exon II and exon V, with three unusual spliced regions of nucleotides from exon III, intron C, and exon IV tandemly interspersed (Fig. 1A). Analysis of potential consensus splice sites in the TFPI-2 gene (GenBank accession no.AF217542) utilized for the generation of asTFPI-2 using splice site predictor [39] revealed no consensus splice sites for subsequent insertion of the observed nucleotide sequences of exon III, intron C and exon IV between exons II and V (Fig. 1C). It is, therefore, highly likely that asTFPI-2 is an aberrant splice product generated from TFPI-2 pre-mRNA in which the splicing of pre-mRNA is misdirected and does not occur solely at *de facto *splice sites normally used by the major, U2-dependent spliceosome [25].

In order to assess the relative levels of the TFPI-2 and asTFPI-2 transcripts, we used semi-quantitative RT-PCR and quantitative real-time PCR and observed a dramatic difference in the levels of these two transcripts in normal cells and tumor cells. Quantification of TFPI-2 mRNA and asTFPI-2 levels using real-time PCR was found to be highly reproducible using transcript-specific primers, with low inter-assay variations. By this procedure, normal cells synthesize ~10-fold more wild-type TFPI-2 transcripts than tumor cells, whereas tumor cells synthesized 4–50 times more asTFPI-2 than normal cells. Moreover, the proportion of asTFPI-2 to that of wild-type TFPI-2 was 7–12-fold higher in tumor cells, while in normal cells, the asTFPI-2 transcript was very low to negligible (Fig. 2 and 3).

An unusual feature of the asTFPI-2 is its lack of detectable 5' – and 3' UTRs. As expected, both 5' and 3'-RACE analyses of total RNA from either normal tissue (placenta) or a tumor sample (lung tumor) resulted in a single amplification product containing the 5'-UTR and 3' – poly (A)^+ ^tail regions of wild-type TFPI-2 cDNA. However, no additional RACE amplification product(s) with either a 5'-UTR or a 3'-poly (A)^+ ^tail was observed in either lung tumor RNA or placental RNA, indicating that the asTFPI-2 transcript lacked these two elements. In addition, application of HUVEC and Colo-205 total RNA samples to oligo-dt columns with subsequent elution of the poly (A)^+ ^RNA and RT-PCR analyses of these elutes resulted exclusively in the amplification of the full-length TFPI-2 transcript (data not shown). Thus, either by message enrichment, RACE analyses and primer walking experiments, we conclude that asTFPI-2 does not contain either a 5'-UTR or a 3'-poly (A)^+ ^tail. Moreover northern blot analysis of total RNA obtained from Colo-205 cells revealed that only the asTFPI-2 transcript (~300 bp) was observed in these cells, further validating the RT-PCR results (Fig. 5). In contrast, two high molecular weight transcripts (1.2 kb and 1.8 kb) for full length TFPI-2 were observed in HUVECs, confirming the results of previous studies [19].

After treating cells with actinomycin D, the TFPI-2 mRNA exhibited a half-life of ~8 h in HUVECs, whereas the asTFPI-*2 *mRNA exhibited a half-life value of ~16 h in Colo-205 cells (Fig. 4). Although it is difficult to directly compare the half-lives of these two transcripts made in normal and transformed cells, the relatively long half-life of the asTFPI-2 transcript is remarkable when one considers that it lacks both a 5'-UTR and 3'-poly (A) tail, two elements that usually offer some protection from exonucleolytic decay [26]. Inasmuch as asTFPI-2 is apparently not translated and exhibits a relatively long half-life, it is tempting to speculate that this splice variant plays some role in tumor homeostasis and studies to address this possibility are ongoing in our laboratory.

Our results are consistent with several reports on differential alternative splicing between cancer and normal expressed sequence tag libraries where there appears to be a higher proportion of disrupted spliced variants in tumor suppressor genes than in non-cancer-related genes [27-29]. In this regard, aberrant splicing has been reported for mdm2, TSG101 and FHIT pre-mRNAs [25,30], exclusively in tumors, and the splicing was characterized by either losses of individual exons, or loss of exons with insertions of unknown DNA sequences without regard for proper splice junctions [31]. Increased aberrant splicing also represent an additional mechanism for the reduction of the amount of wild type tumor suppressor mRNA without mutation, as shown for the NF1 gene [32], or alternative splicing, as shown for a brain-specific exon in *Bin1 *eliminating the activity of the tumor suppressor in melanoma [33]. In addition, several cases of aberrant and alternative splicing of different proteins such as transcription factors, signal transducers and extra-cellular matrix components have been related to tumor cell growth [34]. As a consequence, a number of interventions that either alter or exploit alternative splicing are currently under active investigation for cancer therapy [35,36]. Conceivably, detection of asTFPI-2 could find application in tumor diagnosis, or possibly serve as a marker for cancer progression.

Our laboratory, as well as others, have previously reported that overexpression of candidate tumor suppressor TFPI-2 gene in several highly metastatic tumor cells markedly inhibits their growth and metastasis *in-vivo *by regulating pericellular extracellular matrix (ECM) remodeling and angiogenesis [4-10]. Conversely, hypermethylation of tumor suppressor genes is a major epigenetic change that contributes to tumor progression and is correlated with transcriptional silencing. Hypermethylation of the TFPI-2 gene promoter has been described recently as a possible mechanism for gene silencing in a limited number of tumor cell lines [3,10-12,37]. However, the prevalence of hypermethylation of the TFPI-2 promoter in primary tumors has not been systematically investigated. In many cancer cells, the hypermethylation of the TFPI-2 promoter does not appear to be the sole basis for TFPI-2 gene silencing, as one or more components of the cellular transcription machinery that regulates TFPI-2 expression may also be hypermethylated leading to their silencing as well [13]. The existence of an aberrantly-spliced variant of TFPI-2 that is untranslated, and appears to be highly upregulated in tumor cells, suggests an additional, novel mechanism for further downregulation of TFPI-2 expression in tumor cells.

## Conclusion

The results obtained in this study strongly suggest an additional mechanism in tumor cells to down regulate TFPI-2 expression by aberrant splicing. The existence of a novel TFPI-2 transcript predominantly in tumor cells has not been previously reported. While the present study suggests a direct correlation between the production of a novel TFPI-2 transcript and tumor development, further studies are needed to determine whether the alternative splicing of TFPI-2 pre-mRNA reported here is associated with tumor progression.

## Methods

### Materials

The human tumor cell lines J-82 (bladder cell carcinoma), Capan-2 (pancreatic adenocarcinoma), HepG2 (hepatocellular carcinoma), HT-1080 (fibrosarcoma), Colo-205 (colon adenocarcinoma), and HEK 293 (primary embryonal kidney), were obtained from American Type Culture Collection (Manassas, VA). The human embryonic fibroblast cell line WI-38 was kindly provided by Dr. LVM Rao (University of Texas, Tyler, TX). Human umbilical vein endothelial cells (HUVECs) were obtained from Cambrex (Walkersville, MD). Dulbecco's minimal essential medium (DMEM), penicillin, and streptomycin, actinomycin D and mouse monoclonal anti-tubulin antibody were purchased from Sigma-Aldrich (St Louis, MO). Fetal bovine serum was purchased from Hyclone (Ogden, UT). RNEasy^® ^RNA extraction kit, Oligotex mRNA mini kit, and Plasmid Spin Miniprep kit were purchased from Qiagen (Valencia, CA). Normal human lung, liver and colon RNAs, as well as their corresponding tumor RNAs, were purchased from Chemicon Inc. (Temecula, CA). Human placental total RNA and SMART^® ^RACE kit was purchased from BD Biosciences Clontech (Palo Alto, CA). RT-PCR kit TaqMan^® ^and SYBR^®^-green PCR Master Mix were purchased from Applied Biosystems (Foster City, CA). AccuPrime™ *Pfu *Polymeras and DNase-I were purchased from Invitrogen (Carlsbad, CA). Nylon membrane, Nitrocellulose (NC) membranes and goat anti-rabbit IgG-HRP were purchased from Bio-Rad (Hercules, CA). Random primed labeling kit was purchased from Roche diagnostics (Indianapolis, IN). PCR-Script™ AMP cloning kit and *E. coli *XL1 Blue cells were from Stratagene (Cedar Creek, TX). Chemiluminescent HRP substrate was purchased from Millipore Corporation (Billerica, MA).

### Cell culture and total RNA preparation

All cell lines were maintained in Dulbecco's minimal essential medium (DMEM), supplemented with 10% fetal bovine serum and penicillin-streptomycin. The cells were cultured at 37°C in a humidified atmosphere containing 6% CO_2_, and approximately 5 × 10^6 ^cells were processed for total RNA isolation. The total RNAs were purified using the RNEasy^® ^RNA extraction kit according to the manufacturer's instruction. All purified RNA samples were further digested with to ensure complete removal of any contaminating genomic or mitochondrial DNA. The DNase-I was subsequently inactivated by incubation with 2 mM EDTA at 65°C for 15 min. Total RNA samples from HUVECs and Colo-205 cells were also subjected to further purification on oligo-dt columns (Oligotex) to obtain poly (A)^+ ^RNA.

### Rapid amplification of cDNA 5' and 3'ends (5'-and 3'-RACE)

The corresponding first-strand cDNAs were prepared from 1 μg of total RNA, using the PowerScript reverse transcriptase, 5'-CDS^® ^primer (modified oligo-dT primer) and BD™ SMART IIA^® ^primer using the BD™ SMART^® ^RACE cDNA amplification kit. Dilutions of each 5' and 3' RACE-ready cDNAs were used in PCR amplification reactions with the SMART^® ^RACE kit universal primer mix and either gene-specific antisense exon IV primers (5'-TAC TTT TCT GTG GAC CCC TCAC-3'), to amplify *TFPI-2*, or exon V primer (5'-TTG CTT CTT CCG AAT TTT CCG G-3') to amplify 5' ends of both TFPI-2/asTFPI-2. Alternatively asTFPI-2 specific primer (5'-CTT TTG CGG ATT CAA C-3') was also used in combination with the universal primer mix. The exon II sense primer was used to amplify 3' ends (5'-AAC GCC AAC AAT TTC TAC ACC T-3') in combination with the universal primer mix. The 5' and 3' RACE cDNAs syntheses and PCR amplifications were performed according to the manufacturer's instruction.

### Nucleotide sequencing analysis

A series of oligonucleotide primers spanning from the cap region (FT2-CAP), exon I (FT2-SP1, FT2-Ex1A, FT2-Ex1B, FT2-Ex1C), intron A (FT2-Int1A, FT2-Int1B), exon II (FT2-Ex2), and exon V (RT2-R1) and 3'-UTR (RHT-OD, RT2-PAT), of the TFPI-2 gene were designed to identify the 5' and 3' boundaries of the asTFPI-2 transcript (Table 1). The PCR amplicons obtained from the primer set (FT2-Ex2 as sense and RHT-OD as antisense primers) were ligated into pPCR-Script™ AMP cloning vector and transformed into competent XL1 Blue cells. Plasmid DNAs were prepared from individually selected clones, using a Plasmid Spin Miniprep kit, and vector primers were used to for sequencing the inserts. The nucleotide sequence was determined by the dideoxy chain termination method using the BigDye Terminator Cycle Sequencing Kit in a Model 377 Sequencing System (Applied Biosystems).

**Table 1:** Oligonucleotide primers used to amplify asTFPI-2

**Name of the primer**	**Sequence (5' to 3')**
FT2-CAP	GAA AGC CGC GCA CCT CCT
FT2-SP1	CTG CAC CAT GGA CCC CGC T
FT2-Ex1A	TGT CGA TTC TGC TGC TTT TCC TG
FT2-Ex1B	CTG CAC TGG GCG ATG CTG CT
FT2-Ex1C	CTG CTC AGG AGC CAA CAG
FT2-Int1A	AGC CTC GCT TTC TCC AGG TCC
FT2-Int1B	TAG GAG CTA CGC CTG ACC ACT T
FT2-EX2	AGG AAA TAA CGC GGA GAT CTG TCT
RT2-R1	TTG CTT CTT CCG AAT TTT CCG G
RHT-OD	TTAA AAT TGC TTC TTC CGA AT
RT2-PAT	GTC ATA TTA TTC TTC AGA TAC

### Semi-quantitative RT-PCR of TFPI-2 transcripts

The first strand cDNAs were prepared from 2 μg of total RNA using random hexamers and a reverse transcriptase-PCR (RT-PCR) kit in a total volume of 50 μl. The TFPI-2 and asTFPI-2 transcripts were amplified using a common sense primer (5'-AGG AAA TAA CGC GGA GAT CTG TCT-3') and a common antisense primer (5'-TTAAAATTGCTTCTTCCGAAT-3'). The constitutively expressed endogenous housekeeping gene, cyclophilin A (using sense primer 5'-GTCTCCTTTGAGCTGTTTGC-3' and antisense primer 5'-AAGCAGGAACCCTTATAACC-3') was also amplified in separate tubes. The controlled semi-quantitative RT-PCR assays were performed using 1 μl of first-strand cDNA product and AccuPrime™ *Pfu *polymerase in a total volume of 50 μl. PCR conditions were 1 cycle of 94°C for 2 min; 30 cycles of 94°C for 30 sec (denaturation), 60°C for 30 sec (annealing) and 68°C for 1 min (extension) followed by 1 cycle of 68°C for 5 minutes (final extension). Aliquots of all PCR products were analyzed in 2% agarose gels and visualized with 0.1 mg/ml ethidium bromide staining. The relative intensity of each amplicon was quantified using Quantiscan software (Biosoft™) and intensities are reported in arbitrary units (A.U.). The intensity of each amplicon was normalized to 10,000 A.U. levels of cyclophilin A amplified from each sample.

### Splice variant-specific quantitative RT-PCR

A two-step, quantitative real-time RT-PCR was performed in triplicate using an ABI Prism 7000 Sequence Detection System (Applied Biosystems) with standard temperature protocol and 2x SYBR Green PCR Master Mix reagent in a total volume of 25 μl. The first-strand cDNAs were prepared from 1 μg of total RNA in a final volume of 50 μl using random hexamers and a reverse-transcriptase PCR kit. For absolute quantitation, the PCRScript-TFPI-2 and PCRScript-asTFPI-2 plasmid serial dilutions, reflecting copy numbers (1 × 10^0^-1 × 10^6^), were used to obtain a standard curve correlating the copy number with the threshold cycle number (C_t _values). Primers used for amplification were as follows: TFPI-2, sense (5'-AAC GCC AAC AAT TTC TAC ACC T-3') and antisense (5'-TAC TTT TCT GTG GAC CCC TCAC-3'); asTFPI-2, sense (5'-AAC GCC AAC AAT TTC TAC ACC T-3') and antisense (5'-CTT TTG CGG ATT CAA C-3'). The cDNA products were quantified from each sample in terms of the TFPI-2 and asTFPI-2 copy numbers. As a control, cyclophilin A copy numbers were obtained in a similar manner, and the relative copy numbers of TFPI-2 and asTFPI-2 were calculated after normalizing to 10^2 ^copies of cyclophilin A transcript for each sample. To assure the primers specificity, the amplicons were subjected to melting curve analysis, agarose gel electrophoresis and nucleotide sequencing. Statistical analysis was performed using GraphPad Prism software (San Diego, CA) and a P-value < 0.05 was considered significant.

### Determination of mRNA stability

The relative stabilities of the wild-type TFPI-2 and as TFPI-2 transcripts were assessed in HUVECs and Colo-205 cells, respectively, following treatment of the cells with the transcriptional inhibitor, actinomycin D. Briefly, subconfluent cells were exposed to actinomycin D (5 μg/ml), and the cells harvested for total RNA extraction at selected times (0–24 h) following the addition of actinomycin D [38]. RNA extraction, cDNA synthesis, and splice variant-specific quantitative real-time PCR analysis were performed as described above. The half-lives of TFPI-2 and asTFPI-2 transcript was determined by calculating linear regressions from a log-linear plot of mRNA expression versus time (SigmaPlot).

### Northern Blotting

Total RNA from HUVEC and Colo-205 cells was prepared as described above. RNA (20 μg) was electrophoresed in a 1.2% agarose-formaldehyde gel, and transferred to a nylon membrane overnight by capillary action. The membrane was pre-hybridized at 65°C for 3 h and hybridized at 42°C overnight with an asTFPI-2 cDNA probe labeled with ^32^P-deoxycytidine triphosphate by random primed labeling (Roche) according to the manufacturer's instruction. The membrane was washed sequentially with 2% SSC containing 0.1% SDS for 15 minutes at room temperature, 0.5% SSC containing 0.1% SDS for 20 min at room temperature, and 0.1% SSC containing 1% SDS for 10 min at 65°C. The blot was then exposed to X-ray film at -70°C for 24–72 h.

### Western blot analysis

Various cells (~1 × 10^6^) were lysed by sonication in 500 μl of 125 mM Tris-HCl (pH 6.8) containing 2% SDS, 10% glycerol, 50 mM sodium phosphate, 10 mM benzamidine and 1 μg/ml aprotinin, centrifuged for 15 min at 10,000 × g at 4°C, and the supernatant recovered. The supernatants were boiled for 3 min and subjected to SDS-PAGE in 4–20% polyacrylamide gradient gels. Following electrophoresis, the proteins were electrotransferred to nitrocellulose (NC) membranes and subsequently blocked with 5% calf serum/TBS/0.1% Tween 20 at 37°C for 2 h. The NC membranes were then probed with rabbit anti-TFPI-2 IgG [21], washed, and incubated with diluted goat anti-rabbit IgG-HRP. Immunoreactive proteins were identified using a chemiluminescent reagent system. Separate blots were treated with mouse monoclonal anti-alpha tubulin antibody in order to demonstrate equal loading of the proteins in the gel.

## Competing interests

The author(s) declare that they have no competing interests.

## Authors' contributions

P.K., H.S.C., and W.K. designed the research; P.K. discovered asTFPI-2 and performed the research; P.K., H.S.C., and W.K. analyzed the data; P.K. and W.K. wrote the manuscript.
